# Does school reopening affect SARS-CoV-2 seroprevalence among school-age children in Milan?

**DOI:** 10.1371/journal.pone.0257046

**Published:** 2021-09-02

**Authors:** Lucia Barcellini, Federica Forlanini, Arianna Sangiorgio, Greta Gambacorta, Luisella Alberti, Andrea Meta, Paola Gaia, Antonella Amendola, Elisabetta Tanzi, Valentina Massa, Elisa Borghi, Valentina Fabiano, Gian Vincenzo Zuccotti

**Affiliations:** 1 Department of Paediatrics, Children Hospital V. Buzzi, University of Milan, Milan, Italy; 2 Newborn Screening Laboratory, Regional Reference Center for Metabolic Screening, Buzzi Children Hospital, Milan, Italy; 3 Department of Biomedical Sciences for Health, University of Milan, Milan, Italy; 4 Department of Health Sciences, University of Milan, Milan, Italy; St George’s University of London, UNITED KINGDOM

## Abstract

The benefits of schools’ closure, used as a containment strategy by many European countries, must be carefully considered against the adverse effects of child wellbeing. In this study, we assessed SARS-CoV-2 seroprevalence, which better estimates the real extent of the infection unraveling asymptomatic cases, among schoolchildren aged 3 to 18 in Milan, using dried blood spot, a safe and extremely viable methods for children, and then compared it between September 2020 and January 2021. Secondly, we evaluated the seroconversion rate and compared it between students attending schools in presence and those switched to distance-learning, using a logistic regression model, both as univariate and multivariate, adjusting for age and biological-sex. Among 1109 pupils, we found a seroprevalence of 2.8% in September before school reopening, while in January 2021, the seropositive rate was 12.5%, reflecting the general growth rate of infections during the second pandemic wave. The overall seroconversion rate was 10%, with no differences based on biological-sex and age groups; we observed no seroreversion. When considered age groups, the seroconversion rate was 10.5% (95%Confidence Interval, 2.9–24.8) among children attending preschools, 10.6% (95%Confidence Interval, 8.2–13.4) for primary schools, 9.9% (95%Confidence Interval, 6.8–13.8) for secondary schools, and 7.8% (95%Confidence Interval, 4–13.2) among high-school students. Interestingly, no differences in seroconversion rate were found between students who attended school compared to those who started remote learning in the first days of November. Furthermore, most patients (61%) reported that the contact occurred within the household. We reported a low seroconversion rate among school children in Milan, with no differences between those who attended from September 2020 to January 2021 compared to those who switched to remote learning in the first days of November. Our data suggest that schools do not amplify SARS-CoV-2 transmission, but rather reflect the level of the transmission in the community.

## Introduction

Proactive school and daycare facilities closure is one of the presumed key effective strategies to limit virus spread into the community. Contact-tracing investigations, outbreaks surveillance, and observational epidemiological data suggest that school re-opening has not been associated with significant increase in community transmission, when appropriate mitigation measures were applied, although assessing the effectiveness of school closures, compared with other nonpharmaceutical interventions (NPIs), is highly challenging [1]. Besides, the benefits of closure must be carefully considered against the adverse effects of child wellbeing.

Many European countries, including Italy, closed schools during the first wave of COVID-19 as a containment strategy. In Lombardy, one of the most affected regions of Italy, after 6 months of closure, schools fully reopened in mid-September 2020. However, due to the escalating number of new cases, on the 6th of November, middle and high schools were closed again, switching to distance learning.

The aim of the study is to assess SARS-CoV-2 seroprevalence among pupils and characterize differences in transmission dynamics between students attending schools in presence and those switched to distance-learning.

## Materials and methods

### Study plan

We implemented a prospective multicenter SARS-CoV-2 serologic testing program across 15 different Institutes, representative of different territorial areas of Milan. Recruitment was organized through awareness campaigns at school and study participation was completely voluntary. Subjects were eligible if aged from 3 to 18 years old. The study was approved by the ASST-FBF-Sacco Institutional Review Board, it is conformed to the principles embodied in the Declaration of Helsinki, so informed consents were obtained from all parents. The participants received a collection kit: a safety lancing needles, PKI 226 filter paper (PerkinElmer, USA), bandage, and the protocol for home testing. A tutorial video of dried blood spots (DBS) sampling, performed by trained nurses was available on schools/hospital website. Samples were collected before school re-opening (7-14^th^ September) and in January 2021 (15-29^th^). All samples were self-collected by the participants, gathered by the schools, and sent to the Newborn Screening Laboratory, Regional Reference Center for metabolic screening, of Buzzi Children Hospital. All seropositive patients were given a molecular nasopharyngeal swab at the first positive finding and were asked to answer to a questionnaire covering sociodemographic and epidemiological information (S1 File).

### Serological testing for SARS-CoV-2

Filter papers were tested by a fully automated GSP^®^/DELFIA^®^ anti SARS-CoV2 kit (PerkinElmer) targeting IgG antibodies against SARS-CoV-2 Spike protein, which had a sensitivity of 96% and a specificity of 100% [2]. Tests were performed as per manufactured instructions [2]. Dried blood spot sample, as well as being minimally invasive and more accepted by patients, is a proven population-based screening method and recent studies showed a good concordance between results obtained with DBS and serum samples [3,4]. Results were classified according to ratio values of sample absorbance over calibrator (ODs/Cal), as recommended by the manufacturer, into three categories: negative (<0.9), borderline (≥0.9 and <1.19), and positive (≥1.19).

### Statistical analysis

First, we assessed the seroprevalence in all participants based on antibody detection, next we evaluated the seroconversion rate, and compared them among the two time periods, using a logistic regression model, both as univariate and multivariate analysis, adjusting for age and biological-sex. Statistical significance was set at p<0.05. Statistical analysis was performed with STATA 16.1.

## Results

A total of 2646 tests were initially collected from 1323 patients. We excluded 214 (16.1%) patients, because for 13 (0.9%) of them the results were borderline/inconclusive and not repeated at, at least, one of the two timepoint, and for 201 (15.2%) the samples were insufficient/inadequate to perform the analysis. Hence, we analyzed the results of 1109 patients. Characteristics of study population are presented in Table 1.

**Table 1:** Characteristics of study subjects by two time period and serologic results.

		September 2020			January 2021		
		Total	Negative	Positive	Total	Negative	Positive
**N (%, 95% CI)**		1,109	1,078 (97.2%, 96–98)	31 (2.8%, 1.9–3.9)	1,109	970 (87.5%, 85–89)	139 (12.5%, 10.6–14.6)
**Ages and stages**	3–5 years	70 (6.3%)	69	1	39 (3.5%)	34	5
	6–10 years	608 (54.8%)	588	20	592 (53.4%)	513	79
	11–13 years	298 (26.9%)	292	6	320 (28.9%)	281	39
	14–18 years	133 (12.0%)	129	4	158 (14.2%)	142	16
**Biological sex**	Male	578 (52.1%)	563 (52.2%)	15 (48.4%)	578 (52.1%)	509 (52.5%)	69 (49.6%)
	Female	531 (47.9%)	515 (47.8%)	16 (51.6%)	531 (47.9%)	461 (47.5%)	70 (50.4%)
**Serologic lab value,** ratio		0.3 (0.2–0.3)	0.3 (0.2–0.3)	3.7 (2.75–5.85)	0.2 (0.2–0.3)	0.2 (0.2–0.3)	7.8 (3.4–12.8)
**NPS post-positive serology**	Not performed				23 (16.5%)		23 (16.5%)
	Positive				3 (2.2%)		3 (2.2%)
	Negative	31 (100%)		31 (100%)	113 (81.3%)		113 (81.3%)

Abbreviations: IQR, interquartile range; NPS, Nasopharyngeal Swab.

In September 2020, 31 out of 1109 were positive, corresponding to a seroprevalence of 2.8% (95%confidence interval, CI, 1.9–3.9%), while in January 2021, the seropositive rate was 12.5% (95%CI, 10.6–14.6%).

Of the seropositive patients, 115 (82.7%) participated to the questionnaire: 69 (60%) patients stated a known history of contact with a confirmed COVID-19 patient, with most contacts reported in October 2020 (30%) and November 2020 (38%). Moreover, for 43(61%) patients the contact happened within the household, while at school only for 26 (37%). Seventy-eight (67.8%) seropositive students never experienced SARS-CoV-2 related symptoms. Of the students with a positive serologic result, only 3 (2.2%) had a positive nasopharyngeal swab for SARS-CoV-2, and all were asymptomatic at that time.

A total of 108 (10%) children showed a seroconversion between the two timepoints; we observed no seroreversion. The seroconversion rate was similar between males and females (p-value = 0.62), and between age classes (p-value>0.05). When considered age groups, the seroconversion rate was 10.5% (95%CI, 2.9–24.8) among children attending preschools, 10.6% (95%CI, 8.2–13.4) for primary schools, 9.9% (95%CI, 6.8–13.8) for secondary schools, and 7.8% (95%CI, 4–13.2) among high-school students.

As, in November, pupils attending pre-schools and primary schools (aged 3–12 years) could attend, while secondary schools (aged 13–18 years) were switched to distance learning, we investigated whether this measure affected the seroconversion rate in the two groups, as showed in Table 2.

**Table 2:** Characteristics of pupils who attended in presence and who switched to remote learning.

		3–12 years old				13–18 years old			
		September 2020		January 2021		September 2020		January 2021	
		Negative	Positive	Negative	Positive	Negative	Positive	Negative	Positive
		N = 869	N = 25	N = 743	N = 115	N = 209	N = 6	N = 227	N = 24
**Age,** median years (IQR)		9 (7–10)	9 (7–9)	9 (7–11)	9 (8–11)	14 (13–16)	14.5 (13–15)	14 (13–16)	14 (13–15)
**Biological sex**	Male	451 (51.9%)	10 (40.0%)	383 (51.5%)	55 (47.8%)	112 (53.6%)	5 (83.3%)	126 (55.5%)	14 (58.3%)
	Female	418 (48.1%)	15 (60.0%)	360 (48.5%)	60 (52.2%)	97 (46.4%)	1 (16.7%)	101 (44.5%)	10 (41.7%)
**Serological lab value,** ratio		0.3 (0.2–0.3)	3.5 (2.6–5.85)	0.2 (0.2–0.3)	7.9 (3.6–13)	0.3 (0.2–0.3)	4.75 (4.15–28.65)	0.2 (0.1–0.3)	5.27 (2.5–9.9)
**NPS post-positive serology**	Not performed				21 (18.3%)				2 (8%)
	Positive				3 (2.6%)				
	Negative		25 (100%)		91 (79.1%)		6 (100%)		22 (92%)

Abbreviations: IQR, interquartile range; NSS, Nasopharyngeal Swab.

Between September 2020 and January 2021, the seroconversion rate among children aged 3 to 12 years was 10.8% (95%CI, 8.7–13.1), while in the age group 13–18 it was 7.3% (95%CI, 4.4–11.3), showing no differences both at univariate (p-value = 0.11; Odds Ratio (OR) 0.65, Standard Error (SE) 0.17, 95%CI 0.38–1.1) and multivariate analysis (p-value = 0.20; OR 0.55, SE 0.26, 95%CI 0.22–1.4).

## Discussion

Our study shows a SARS-CoV2 seroprevalence among pupils of 2.8% in September 2020 before schools reopening, and a seroprevalence of 12.5% in January 2021. The low seroprevalence found in September is concordant with what reported by several studies during the first pandemic wave, which show a smaller proportion of seropositive children compared to adults, around 1–10%. [1] The increase in schoolchildren seroprevalence in January 2021 reflects the general growth rate of infections during the second pandemic wave.

We found an overall seroconversion rate of 10%, with no differences based on biological-sex and age groups. Considering students who attended schools in presence versus those switched to e-learning, we found no differences neither in seroprevalence nor in the seroconversion rate. The role of school-based transmission in the epidemiology of SARS-CoV-2 is not fully elucidated. Some modeling studies indicated that schools closure is associated with reduced SARS-CoV-2 transmission in to the community, with the biggest impact achieved by reducing contacts in secondary schools [5–7]. However, it is challenging to isolate the real impact of proactive schools’ closure as an independent NPIs, since this measure has been often introduced with other mitigation actions, such as workplace closure, remote work policies, large-scale lockdowns with internal travel banned and reduction of social mixing activities. On the contrary, data from epidemiological surveillance, outbreaks and cluster studies indicate that the transmission within school children is low [8,9]. The European Centre for disease control and prevention (ECDC) surveillance data suggest that the re-opening of schools in mid-September 2020 was not temporally associated with an increase in case rates among children. Moreover, a large prospective study by Public Health England national surveillance concludes that the SARS-CoV-2 outbreaks in educational settings were uncommon and their number was strongly associated with regional COVID-19 incidence [10]. Our results are in agreement, questioning the role of school re-opening as a driver of the second COVID-19 wave and of the increased transmission into the community. Nevertheless, all the existing current evidence about transmission dynamics within educational settings derive from contact tracing studies, modeling studies and cluster investigations. This study has some limitation. First, we could not track the transmission dynamics effectively being this only a serological study, but only assess the seroprevalence and the seroconversion rate. Furthermore, as already anticipated, it was not possible to estimate the impact of other NPIs that could have limited transmission in the school environment, since all the preventive measures for physical distancing have been applied in all the Institutes, such as mandatory wearing of surgical mask, single desks, and temporal and special pathways for different classes. Finally, it is important to underline that, when a case was identified within a class, all children and staff involved were quarantined, so also this measure limited further transmission. On the opposite, the strength of our study is investing, in a cohort of 1109 pupils, the seroprevalence, which better estimates the real extent of the infection, unraveling asymptomatic cases, that in children account for up to the 28% of cases [11]. In fact, a key limitation in understanding the real extent of transmission among children is their being frequently asymptomatic. Moreover, case identification has been often limited by capacity gaps in testing or reluctance to test young children with nasopharyngeal swabs. Therefore, seroprevalence studies, like ours, may facilitate the evaluation of infection rates, promoting an informed policy on the management of the educational setting.

## Conclusion

We reported a low seroconversion rate among school children in Milan, with no differences between those who attended from September 2020 to January 2021 compared to those who switched to remote learning in the first days of November. Our data suggest that schools do not amplify SARS-CoV-2 transmission, but rather reflect the level of the transmission in the community.

## Supporting information

S1 File Questionnaire: Questionnaire administered to positive patients. (DOCX)

S1 Dataset Anonymized dataset. (XLSX)
