# Supplementary material for: Does school reopening affect SARS-CoV-2 seroprevalence among school-age children in Milan?
Source: PLoS One. 2021 Sep 2;16(9):e0257046. doi: 10.1371/journal.pone.0257046 (PMC8412254; doi:10.1371/journal.pone.0257046)
Supplement: S1 File — (DOCX) [file pone.0257046.s001.docx]

**S1 File: Questionnaire**

| **Questionnaire** | |
| --- | --- |
| First name  Last name | ___________________________  ___________________________ |
| DOB | ___/___/___ |
| Attended schools  Grade level | ___________________________  ___________________________ |
| Did you participate in presence?  If so, until when? | □ Yes □ No  ___/___/___ |
| Do you have any brothers or sisters?  Did your brother / sister attend in presence?  If so, until when? | □ Yes □ No  □ Yes ì □ No  ___/___/___ |
| Did your parents work remotely in smart working mode? | □ Yes □ No |
| How do you get to school? | □ Public transportation  □ Walking  □ By car  Others___________ |
| Have you had any contact with a confirmed case of COVID-19? | □ Yes □ No  If so, when? ___/___/___  If so, what kind of contact?  Relatives □  Classmate □  Others____________ |
| Did your family members have symptoms compatible with COVID-19 infection? | □ Yes □ No  If so, when? _________ |
| Did any of your classmates have symptoms compatible with COVID-19 infection? | □ Yes □ No  If so, when? _________ |
| Have you ever performed a nasopharyngeal swab for COVID-19? | □ Yes □ No  If so, when? _________ |
| If so, what was the result? | □ Positive □ Negative |
| Have you ever performed COVID-19 serology before this? | □ Yes □ No  If so, when? _________ |
| If so, what was the result? | □ Positive □ Negative |
| Have you ever had COVID19 related symptoms? | □ Yes □ No  If so, when?  From _________  To _________ |
| Fever ≥38°C | Rash |
| Fatigue | Anosmia/ageusia |
| Sore throat | Lack of appetite |
| Cough | Diarrhea |
| Rhinitis | Abdominal pain |
| Dyspnea | Nausea/vomit |
| Have these symptoms required the attention of your physician? | □ Yes □ No |
| Did these symptoms lead to hospitalization? | □ Yes □ No |
